# Extremely high relative growth rate makes the cabbage white, *Pieris rapae*, a global pest with highly abundant and migratory nature

**DOI:** 10.1038/s41598-023-36735-8

**Published:** 2023-06-15

**Authors:** Kotaro Konno

**Affiliations:** Institute of Agrobiological Sciences, National Agriculture and Food Research Organization (NARO), 1-2 Owashi, Tsukuba, 305-8634 Japan

**Keywords:** Ecology, Ecology

## Abstract

The small cabbage white butterfly, *Pieris rapae*, is an extraordinarily abundant migratory pest of cabbage that causes severe damage worldwide without known reasons. I here show that the average relative growth rate (RGR: the ratio of the daily increase of biomass to total biomass) of herbivore (G_h_; an indicator of the growth speed of herbivore) of *P. rapae* on cabbage during the larval period is larger by far than those of all other insect–plant pairs tested. It exceeds 1.15 (/day),—meaning that the biomass more than doubles each day—compared to 0.1–0.7 for most insect–plant pairs, including that of *Pieris melete*, a sibling of *P. rapae* which never becomes a pest of cabbage. My data further showed the RGR in the larval stage (larval G_h_), positively correlates with abundance and/or migratoriness of insect herbivores. These results together with my mathematical food web model suggest that the extraordinarily high larval G_h_ of *P. rapae* is the primary reason for its ubiquitously severe pest status accompanied with its abundance and migratoriness, and that the RGR of herbivores, G_h_, characterizing the plant–herbivore interface at the bottom of the food webs is an important factor affecting whole ecosystems, including animal abundance, fauna size, plant damage levels, competitiveness among herbivorous species, determination of hostplant, invasiveness, and the evolution of animal traits involved in the so-called r/K strategy, such as migratoriness. Knowledge about G_h_ will be crucial to controlling pests and improving the negative effects of human activity on ecosystems including faunal decline (or defaunation).

## Introduction

The determinants of the biomass of animals and size of fauna are not well understood^1–3^. As a general rule in terrestrial ecosystems, the biomass of herbivores, including herbivorous insects, is generally smaller than the plant biomass^1–4^, and thus plant damage by herbivorous insects is also generally small, impacting less than 5% of the total leaf mass annually^5,6^. This status is so-called “green world”, and a number of attempts including the HSS hypothesis^1^ and Konno’s food web model^7^ have been proposed to explain the green world status. Konno’s food web model can be used to predict the absolute biomass of herbivores and carnivores, as well as absolute plant damage. It suggests that the green world necessarily appears in terrestrial ecosystems characterised by high predator efficiency (promoted by high prey visibility) and low nutritive values of plant materials: this results in very limited biomass of herbivorous insects and a very low annual rate of herbivory^7^.

**Figure 1:**
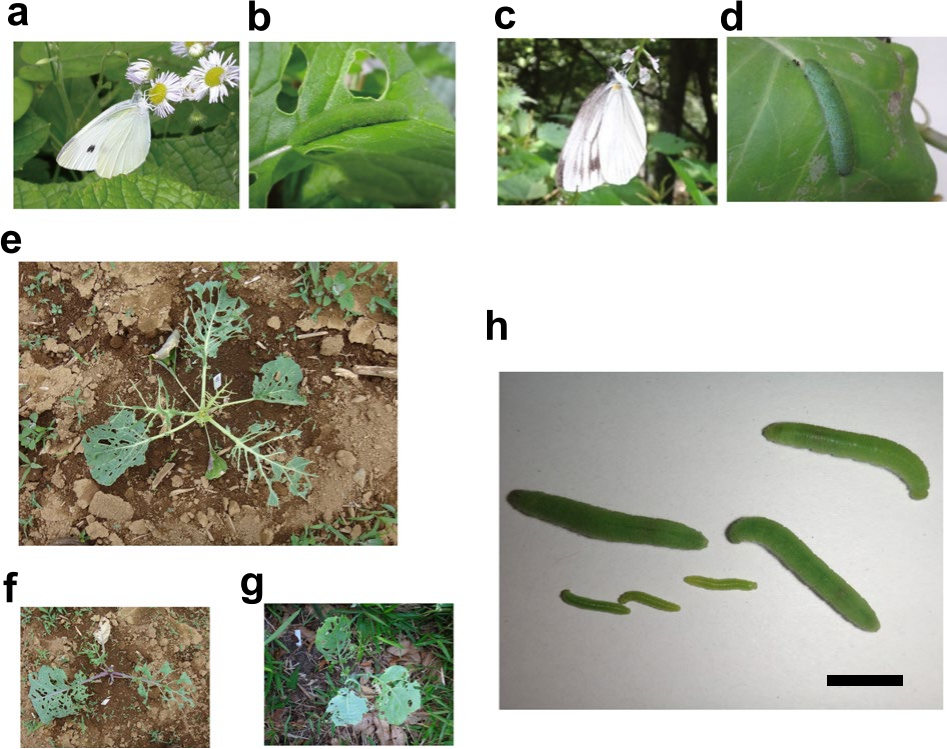
The small white, *Pieris rapae*, its damage to cabbage plants, its extraordinary growth rate (speed), and its sibling species *Pieris melete*. (**a**, **b**) *Pieris rapae*, adult (**a**) and final instar larva (**b**). (**c**, **d**) *Pieris melete*, adult (**c**) and final instar larva (**d**). (**e**–**g**) Damage caused by *Pieris rapae* larvae on *Brassica oleracea*, on round-headed cabbage cultivar, Kinkei-201, grown in cultivated fields over a 15 day period in May–June (**e**), on a wild variety 0-176 grown in cultivated fields over a 14 day period in May–June (**f**), and on a wild variety 0-176 in a forest over a 48 day period in June–July (**g**). (**h**) The extraordinarily high relative growth rate (RGR), or growth speed, of the cabbage white larvae, *Pieris rapae*, feeding on cabbage leaves at 25 ºC. A comparison of larvae 4 days after hatching (i.e., small larvae below: average mass 11.4 mg) to larvae 8 days after hatching (large larvae: average mass ≥ 214.9 mg) shows that *P. rapae* larvae can grow 18.8 times in mass in only 4 days, which means they can more than double their mass each day. The bar indicates 1 cm.

The small cabbage white butterfly, *Pieris rapae* (Lineaeus, 1758; Fig. 1a, b), is an apparent exception to the green world status, due to severe damage caused by larvae on cabbage. It is arguably one of the most common butterflies in temperate regions around the world, including those in Europe, North America, North Asia, East Asia, Australia, and New Zealand^8–12^. Indeed, *P. rapae* is so abundant that it often outnumbers all other butterflies combined in many northeastern states in the USA^9^. A recent study clarified that *P. rapae* originated in the Mediterranean and West Asian regions, invaded East Asia from Western Asia in the year 1200 BP, and spread to the rest of the world, including North America, Australia and New Zealand, over the last 160 years^8^. It is reported to be highly migratory^13,14^, capable of migrating across the Pyrene Mountains^15^ and Mediterranean Sea^16^ in large numbers. This exceptional migration capacity may contribute to its invasion. Further, *P. rapae* not only invaded successfully but also became a major pest of cabbage and other cruciferous crops, and is currently the most abundant cabbage pest worldwide^8^, including in Canada^17^ and Japan^18^. *P. rapae* is notorious for causing severe damage to cultivated cabbage (Fig. 1). In the absence of control measures, total defoliation is not uncommon, and can result in 80% of the cabbage heads becoming unmarketable^17^. In spite of the globally severe pest status of *P. rapae*, there has been no reasonable explanation for its exceptional ecology and pest status.

In the present study, I aimed to find the reason for the severe pest status of *P. rapae* larvae against cabbage from experimental and theoretical approaches and obtained the results indicating that the extremely large relative growth rate (RGR) of herbivore (G_h_) of *P. rapae* larvae feeding on cabbage is the major factor contributing to the severe pest status of *P. rapae* larvae through increasing abundance, competitiveness and migratoriness of *P. rapae* larvae. Further effects of G_h_ on whole ecosystems including faunal size will be discussed.

## Results

### Severe herbivory on cabbage by the small white butterfly larvae

Since most studies on cabbage damage caused by *P. rapae* have been performed in an agricultural context, there are only very limited cabbage herbivory data comparable to the data on the strength of other herbivories from other ecological studies, in which damage is generally expressed as the % leaf area damage per unit time or per well-defined time period. However, I observed very high levels of herbivory, which were constant throughout the growing season (31.2 ± 17.5 (average ± standard error) % leaf area/20–22 days in May–June (n = 6) and 34.3 ± 5.3% leaf area/34–49 days in September–November (n = 7)) in a round-headed cabbage cultivar, Kinkei201, grown in cultivated fields (Fig. 1e). These high levels of herbivory of cabbage by *P. rapae* (around 1/3 of the leaf area was eaten in 1–2 month period) were in sharp contrast with the low levels of herbivory (mostly around 5%/year) of the leaves of plants in a variety of terrestrial systems, such as forests and grasslands of tropical, temperate and sub-arctic ecosystems^5–7^. The high level of herbivory on cabbage was neither attributable to the cabbage individuals being from a cultivated variety nor because the tests were performed in cultivated field (artificial conditions), since a wild variety of cabbage from the Mediterranean region (0–176) also suffered severely from the damage under both field and forest (wild) conditions (50.5 ± 16.3% leaf area/20–22 days in May–June (n = 6) and 32.6 ± 6.6% leaf area/34–49 days in September–November (n = 7) in the field, and 55.0 ± 12.2% leaf area/48 days in June–July (n = 2) in the forest), and cabbage cultivars also suffered severe damage in the forest (85.0 ± 5.8% leaf area/48 days in June–July (n = 3)). In addition, a lack of natural enemies in the original and invaded regions is not likely to be the reason for the global pest status of this insect, since in both the original and invaded regions, a number of generalist predators such as ants, spiders, wasps, birds, and frogs, as well as specialist parasitoids such as *Cotesia glomerata*, and viral diseases are commonly observed^19–21^. What, then, can explain the high level of herbivory on cabbage by the small white butterfly?

### Measurement and comparison of relative growth rate (RGR) of herbivore, G_h_, among various insect herbivore–plant pairs

Konno’s food web model^7^ can predict **h**, the absolute biomass of herbivores (as protein) per unit volume of ecosystem, and **c**, the absolute biomass of carnivores (as protein) per unit volume of ecosystem, in convergent equilibrium as follows:

1 $${\mathbf{h} \text{ = }} \frac{{{\text{n}}_{{\text{c}}} \left\{ {\left( {{1} - \upalpha_{{\text{c}}} } \right){\text{P}}_{{{\text{cc}}}} {\text{G}}_{{\text{h}}} {\text{ + 2d}}_{{\text{c}}} {\text{P}}_{{{\text{hc}}}} } \right\}}}{{2\upalpha_{{\text{c}}} {\text{P}}_{{{\text{hc}}}}^{{2}} {\text{S}}}}\;\;\left( {{\text{kg/m}}^{{3}} } \right)$$

2 $${\mathbf{c}} = { }\frac{{{\text{n}}_{{\text{c}}} {\text{G}}_{{\text{h}}} }}{{{\text{P}}_{{{\text{hc}}}}^{{}} {\text{S}}}}\;\; \left( {{\text{kg}}/{\text{m}}^{{3}} } \right)$$

where n_p_, n_h_, and n_c_ (kg/m^3^ or kg protein/m^3^) are the nutritive values of plants (plant material), herbivores (primary consumers), and carnivores (secondary consumers), respectively; P_hc_ and P_cc_ are the preying probability of a carnivore once a predator meets an herbivore and carnivore (as a ratio without a unit); S (/day) is the searching efficiency of carnivores (the ratio of the volume of total ecosystem carnivores can search in a day to the total volume of the carnivores themselves); α_c_ (a ratio without a unit) is the absorption efficiency of carnivores feeding on animal biomass (herbivores and carnivores); and d_c_ (a ratio without a unit) is a decreasing constant determined as the ratio of the biomass (as protein) consumed by carnivores in the form of respiration, metabolism, and excretion to the biomass of the carnivores themselves. Finally, G_h_ (/day), an important factor that I focus on in this study, represents the relative growth rate (RGR) of herbivores. Although several definitions of RGR are available in the literature, here I define RGR as the ratio of the increase of biomass per day to the total biomass of herbivores feeding on plants, following the definition used in several previous studies^7,22,23^.

Equation (1) means that the biomass of herbivores, **h**, is positively correlated with the relative growth rate of herbivore, G_h_, and that when G_h_ is large, **h** is proportional to G_h_. From Eq. (1), PDM_v_, the plant damage intensity, which is the amount (volume) of plant damage per unit volume of ecosystem per day, can be expressed as

3 $${\text{PDMv}} = { }\frac{{\mathbf{h}{\text{e}}_{{\text{h}}} }}{{{\text{n}}_{{\text{h}}} }}{ } = \frac{{{\text{e}}_{{\text{h}}} {\text{n}}_{{\text{c}}} \left\{ {\left( {{1} - {\upalpha }_{{\text{c}}} } \right){\text{P}}_{{{\text{cc}}}} {\text{G}}_{{\text{h}}} + {\text{2d}}_{{\text{c}}} {\text{P}}_{{{\text{hc}}}} } \right\}}}{{{{2\upalpha }}_{{\text{c}}} {\text{P}}_{{{\text{hc}}}}^{{2}} {\text{n}}_{{\text{h}}} {\text{S}}}} \left( {/{\text{day}}} \right).$$

Equation (3) means that PDMv is positively correlated with e_h_ × G_h_, and is proportional to e_h_ × G_h_ when G_h_ is sufficiently high. If the feeding speed of herbivore e_h_ is proportional to G_h_, plant damage PDM_v_ is estimated to be roughly proportional to G_h_^2^ when G_h_ is sufficiently high. Thus, the model raises the possibility that the constant abundance of *P. rapae* and constantly high level of damage to cabbage caused by *P. rapae* larvae may have resulted from the large G_h_ of *P. rapae* larvae.

In order to examine this possibility, I measured average G_h_ during the whole larval stage from egg hatching to pupation at 25 °C in various insect herbivore (mostly lepidopteran larvae including several pest species)–host plant pairs. Average daily G_h_ during the larval stage (larval G_h_) was calculated as follows using the pupal mass, egg mass, and duration of larval stage (days):

4 $${\text{Larval }}\;{\text{G}}_{{\text{h}}} \; = \;{\text{ exp}}_{{{1}0}} \left[ {{\text{log}}_{{{1}0}} \left\{ {{\text{pupal }}\;{\text{mass}}/{\text{egg }}\;{\text{mass}}} \right\}/\{ {\text{duration}}\; \, \left( {{\text{days}}} \right)\;{\text{ of }}\;{\text{larval }}\;{\text{stage}}\} } \right]{-}{1}\;\;\; \left( {/{\text{day}}} \right)$$

When calculating larval G_h_, I did not use the mass of larvae as in previous studies. This is because the larvae can include large (up to 80%) and variable amounts of food materials and digestive fluid in their digestive tracts, and thus the measurement of larvae mass may include significant sources of fluctuation. Instead, I calculated larval G_h_ by using the mass of eggs and pupae, which does not include such sources of fluctuation, and thus obtained consistent, reliable, and comparable values of larval G_h_. Larval G_h_ is supposed to be a good assimilation of G_h_ throughout whole life stages because most herbivorous insects grow during the larval stage and not during other stages (egg, pupal, and adult stages).

Larval G_h_ calculated as in Eq. (4) turned out to be an indicator that is surprisingly diverse and distinct among insect herbivore–plant pairs but is fairly constant within each insect herbivore–plant pair with small SE (and also SD) (Table 1). The larvae of *P. rapae* grew extremely quickly on cabbage leaves (Fig. 1f): within an average of 10.5 days, an egg with mass of 0.07 mg hatched into a larva and became a pupa with a mass of 208.5 mg (a 2979-fold increase of mass) (Table 1). The larval G_h_ of the *P. rapae* feeding on a cabbage cultivar (1.161) and wild cabbage (1.183) exceeded 1.1, which means the larval mass of *P. rapae* was 2.1 times that of the previous day, and on average the larval mass more than doubled each day. The larval G_h_ of *P. rapae* was by far the largest among the insect–plant pairs studied (Table 1), and was significantly larger than those of any other insect–plant pairs (two-sided t-test,* p* < 0.003). Many insect–plant pairs had larval G_h_ around 0.3–0.6, including insect–plant pairs containing non-migratory species, such as *Bombyx mandarina* (Bombycidae)–mulberry (0.360), *Papilio memnon* (Papilionidae)–*Citrus* (0.345), and *Papilio protenor* (Papilionidae)–*Citrus* (0.289), but also including insect–plant pairs containing some migratory pest species, such as *Agrius convolvuli* (Sphingidae)^24^–sweet potato (0.466). The insect–plant pairs that showed relatively large larval G_h_ exceeding 0.6–0.7 included many common pest species and migratory species that are rare early in the year (spring) but become very abundant late in the year (autumn), such as *Spodoptera litura* (Noctuidae)–cabbage (larval G_h_ = 0.720–0.773, an abundant polyphagous pest, highly migratory^25,26^), *Lampides boeticus* (Lycaenidae)–kudzu flower (larval G_h_ = 0.983, a major pest of legume, highly migratory^27^), and *Papilio machaon* (Papilionidae)–carrot (0.611, a pest of carrot, parsley and fennel, migratory^28^), and some sphingid moth such as *Theretra oldenlandiae* (larval G_h_ = 0.610, a pest of taro and grapes, migratory^29,30^) and *Macroglossum pyrrhosticta* (larval G_h_ = 0.622, highly migratory^31^). In contrast, in cases when the insect–plant pairs show relatively small larval G_h_ values of less than 0.4, the insects are mostly less common, exert only limited damage on plants, are stable in number, and are non-migratory, such as *Bombyx mandarina*, *Papilio protenor*, *Papilio memnon*, and satyrid butterflies. Satyrid butterflies feed on grass and bamboo and are mostly non-migratory with extremely small larval G_h_, and belong to such pairings as *Mycalesis gotama*–*Miscanthus sinensis* (Chinese silver grass) (larval G_h_ = 0.250) and *Lethe sicelis*-*Pleioblastus chino, var chino* (dwarf bamboo) (larval G_h_ = 0.102). In correspondence with its small larval G_h_, *Mycalesis gotama*, which is widely found in rice fields in Japan, always remains a minor pest of rice with low population density, and exerts only minor damage to rice^32^.

**Table 1:** Average larval G_h_ (relative growth rate during the whole larval stage) from hatching to pupation at 25 °C in various insect herbivore–host plant pairs, including the small white–cabbage pair.

Herbivorous insects	Plant	Abundance on the plant (++, +, −) Migratoriness (+, −))	Egg mass (mg)	Pupal mass (mg)	Pupal mass/egg mass ratio	Larval duration (days)	Larval G_h_ (± s. e.) (Relative growth rate during larval stage) (/day)	*n*
*Pieris rapae*, (Pieridae, Lepidptera)	*Brassica oleracea var Capitata* (cabbage, Brassicae) *cultivar Kinkei-201*	++ +	0.07	208.5	2979	10.4	1.161 (± 0.030)	10
*Pieris rapae*,	*Brassica oleracea wild type* 0 176	++ +	0.07	203.9	2913	10.2	1.183 (± 0.021)	13
*Pieris melete*	*Brassicae oleracea*, Kinkei-201	+ +	0.16	204.7	1279	13.1	0.728 (± 0.018)	14
*Spodoptera litura,* (Noctuidae, Lepidoptera)	*Brassicae oleracea*, Kinkei-201	+ +	0.035	217.0	6199	16.2	0.720 (± 0.037)	5
*Spodoptera litura*	*Brassica oleracea*, O-176	+ +	0.035	328.6	9356	16.0	0.773 (± 0.023)	6
*Mamestra brassicae*, Noctuidae, Lepidptera	*Brassica oleracea*, Kinkei-201	+ +	0.125	319.4	2556	24.5	0.380 (± 0.008)	11
*Eurema mandarina* (former *E. hecabe*), Pieridae	*Lespedeza cuneata var. cuneata*, Fabaceae	+ +	0.20	116.6	583	15.1	0.530 (± 0.019)	9
*Colias erate*, Pieridae	*Trifolium repens*, Fabaceae	+ +	0.12	327.8	2732	15.1	0.690 (± 0.023)	7
*Colias erate*	*Trifolium pratense*	+ +	0.12	329.6	2747	24.5	0.385 (± 0.069)	2 (+ 6 dead)
*Anthochalis scolymus*, Pieridae	*Cardamine occulta*, Brassicae, young seed pod	+ +	0.06	131.9	2198	11.5	0.955 (± 0.023)	6
*Papilio xuthus*, Papilionidae, Lepidoptera	*Citrus sudachi*, Rutaceae, young leaves	+ +	0.83	1099.4	1303	19.4	0.447 (± 0.017)	7
*Papilio protenor*	*Citrus sudachi*, mature leaves	− −	1.6	1814.0	1121	28.0	0.289 (± 0.017)	6
*Papilio memnon*	*Citrus sudachi*, mature leaves	− −	2.3	2529.3	1099	24.2	0.345 (± 0.025)	6
*Papilio machaon*	*Daucus carota*, Apiaceae	+ +	0.8	1471.7	1840	15.8	0.611 (± 0.021)	4
*Byasa alcinous*, Papilionidae	*Aristolochia debilis*, Aristrochiaceae	+ +	1.3	1007.6	757	20.2	0.392 (± 0.011)	11
*Graphium sarpedon*, Papilionidae	*Cinnamomum camphora*, Lauraceae	+ +	1.1	1267.2	1229	20.5	0.419 (± 0.019)	6
*Theretra oldenlandiae*、Sphingidae, Lepidoptera	*Causonis japonica* (former *Cayratia japonica*), Vitaceae	+ +	1.2	2389.0	2107	16.3	0.610 (± 0.044)	4
*Theretra japonica*	*Causonis japonica*	− −	1.2	3484.5	2903	20	0.490 (–)	1
*Acosmeryx castanea*, Sphingidae	*Causonis japonica*	− −	2.4	5772.6	2405	20	0.476 (–)	1
*Macroglossum pyrrhosticta*, Sphingidae	*Paederia foetida*, Rubiaceae	+ +	0.7	1356.5	1924	16.0	0.622 (± 0.048)	7
*Neogurelca himachala*, Sphingidae	*Paederia foetida*	+ +	0.6	839.0	1398	13	0.745 (–)	1
*Agrius convolvuli* Sphingidae	*Ipomoea batatas*, *cultivar Beniharuka*, Convolvulaceae	+ +	0.4	4046.4	10,116	24.2	0.466 (± 0.025)	5
*Bombyx mandarina*, Bombycidae, Lepidoptera	*Morus alba*, Moraceae	− −	0.3	258.6	862	22.0	0.360 (± 0.011)	4
*Samia ricini*, Saturniidae, Lepidoptera	*Ricinus communis*, Euphorbiaceae	n.a n.a	1.7	3032.6	1784	23.3	0.378 (± 0.025)	3
*Lethe sicelis*, Satyrinae, Nymphalidae, Lepidoptera	*Pleioblastus chino var chino*, Bambusoidae, Poaceae	− −	0.6	342.0	570	65.7	0.102 (± 0.004)	3
*Mycalesis gotama*, Satyrinae	*Miscanthus sinensis*, Poaceae	− −	0.5	190.1	359	26.4	0.250 (± 0.004)	27
*Lampides boeticus*, Lycaenidae, Lepidoptera	*Pueraria montana* var. *lobata*, Fabaceae, flowers	++ +	0.04	84.3	2108	11.2	0.983 (± 0.026)	5
*Curetis acuta*, Lycaenidae	*Pueraria montana* var. *lobata*, flowers	++ +	0.15	313.0	1565	12.2	0.828 (± 0.014)	5
*Henosepilachna vigintioctopunctata*, Coccinellidae, Coleoptera	*Solanum carolinense*, Solanaceae	− −	0.15	29.9	199	15	0.423 (± 0.002)	5

**Figure 2:**
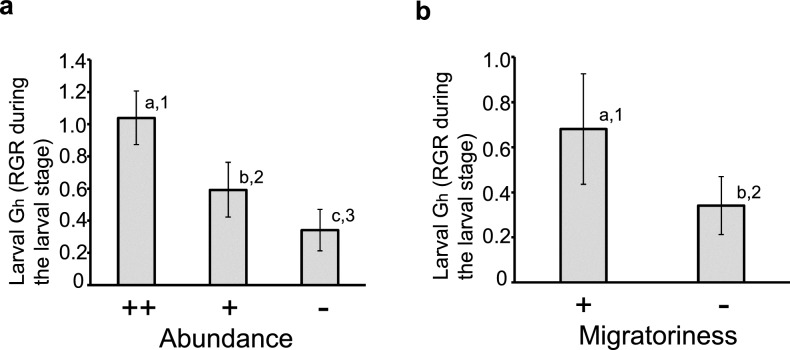
Correlation between larval G_h_ (relative growth rate of herbivorous insects in the larval stage) and ecological traits of herbivorous insects such as abundance and migratoriness. (**a**) Correlation between larval G_h_ and abundance of herbivorous insects. Averages of larval G_h_ were shown for herbivorous insect belonging to following three categories, very abundant (abundance ++, *n* = 4), abundant (abundance + , *n* = 16), and non-abundant (abundance −, *n* = 8). See Table 1 for detail of categorization of insect herbivores and Methods for how insect herbivores were categorized. Error bars indicate standard deviations. Values not followed by the same letters are significantly different (*P* < 0.01; Mann–Whitney *U*-test, pairwise comparison between categories;* P* = 0.0034 between ++ and + , *P* = 0.0066 between ++ and −, *P* = 0.0040 between + and −). Values not followed by the same numbers are significantly different (P < 0.01; Tukey’s test for multiple comparisons). (**b**) Correlation between larval G_h_ and migratoriness of herbivorous insects. Averages of larval G_h_ were shown for herbivorous insect belonging to following two categories, migratory (migratoriness + , *n* = 20), and non-abundant (migratoriness −, *n* = 8). See Table 1 for detail of categorization of insect herbivores. Error bars indicate standard deviations. Values not followed by the same letters are significantly different (*P* < 0.01; Mann–Whitney *U*-test;* P* = 0.005). Values not followed by the same numbers are significantly different (*P* < 0.01; Student’s *t*-test; *P* = 0.0011).

The results shown above suggested that herbivorous insect species with large larval G_h_ have tendency to be more abundant and migratory. In order to clarify this possibility, the herbivorous species shown in Table 1 was categorizes into three groups according to their abundance on host plants, very abundant (abundance ++), abundant (abundance +), and non-abundant (abundance −), and larval G_h_ (average ± SD) of the three groups/categories were compared (Fig. 2a). The larval G_h_ of the very abundant (abundance ++) category was the largest (1.039 ± 0.167, *n* = 4), then that of the abundant (abundance +) category (0.592 ± 0.170, *n* = 16), and that of the non-abundant (abundance −) category was the smallest (0.342 ± 0.129, *n* = 8) and the differences were significant both by non-parametric statistical analysis (*P* < 0.01; Mann–Whitney *U*-test, pairwise comparison between categories;* P* = 0.0034 between ++ and +, *P* = 0.0066 between ++ and −, *P* = 0.0040 between + and −) and by parametric statistical analysis (*P* < 0.0001; Tukey’s test for multiple comparisons). This result indicated that there is correlation between large larval G_h_ and abundance of herbivorous insect species, and that abundant insect herbivores tend to show large larval G_h_ or vice versa.

Similarly, the herbivorous species shown in Table 1 was categorizes into two groups according to their migratoriness on host plants, migratory (migratoriness +), and non-migratory (migratoriness −), and larval G_h_ (average ± SD) of the two groups/categories were compared (Fig. 2b). The larval G_h_ of the migratory (migratorines +) category (0.681 ± 0.246, *n* = 20) was larger than that of the non-migratory (migratoriness −) category (0.342 ± 0.129, *n* = 8) and the differences were significant both by non-parametric statistical analysis (*P* < 0.01; Mann–Whitney *U*-test;* P* = 0.0005) and by parametric statistical analysis (*P* < 0.01; Student’s *t*-test; *P* = 0.0011). This result indicated that there is correlation between large larval G_h_ and migratoriness of herbivorous insect species, and that migratory insect herbivores tend to show large larval G_h_ or vice versa.

Although, above results showed that there is a correlation between abundance of herbivores and large larval G_h_, some exceptional species with small larval G_h_ that are abundant and show outbreak population dynamics do exist. But these species seem to develop traits to escape from natural enemies. For example, *Byasa alcinous* (Papilionidae) (larval G_h_ = 0.392) (Table 1) feeds on toxic *Aristolochia debilis*, and sequesters the toxic component that is unpalatable to natural enemies such as birds^33^, while *Mamestra brassicae* (Noctuidae) (larval G_h_ = 0.380), a major pest of cabbage and other crops^34^, stays underground in the daytime^35^, presumably to escape from predation by natural enemies. In contrast, there are some exceptional species with very large larval G_h_ that are not very abundant, such as *Anthochalis scolymus*, Pieridae (larval G_h_ = 0.955 ± 0.023, *n* = 6). Different from multivoltine *Pieris rapae*, this species, together with other *Anthochalis* species, are strictly monovoltine and have extremely long diapause, one year or more as pupae, and the population loss during the long diapause may possibly explain why *Anthochalis scolymus* is not very abundant.

## Discussion

### RGR of the herbivore, G_h_, as a key ecological factor determining the abundance, competitiveness, invasiveness, and pest status of herbivore, plant damage level, fauna size and faunal decline: observations on *Pieris* species and mechanisms based on mathematical model

*Pieris rapae* showed the highest larval G_h_ among all cabbage-feeding species and Pieridae species tested (Table 1). At the same time, *P. rapae* inflicts the most severe damage to cabbage^8,17,18^, and is by far the most common Pieridae species in Japan and other temperate parts of the world^8–12^, and as far as I know, the *P. rapae*—cabbage relationship is the only or one of the very rare exceptions to the green world status. This correspondence indicates that the large larval G_h_ of *P. rapae* is responsible for its status as the pest most damaging to cabbage. The correspondence between large larval G_h_ and abundance in *P. rapae*, however, is not a mere coincidence confined to *P. rapae*. The statistical analyses of the data shown in Table 1 revealed the general correlation between large larval G_h_ (RGR in larval stage) and abundance of the herbivorous insect species (Fig. 2a). Therefore, it is reasonable to suppose that the large larval G_h_ of *P. rapae* is responsible for the abundance of *P. rapae*. Although the positive correlation between large larval G_h_ and abundance of herbivorous insect is statistically supported for data presented in Table 1, it is still unclear whether the correlation fits in with the Eq. (1) derived from Konno’s food web model^7^. This ambiguity may have resulted from rather qualitative nature of evaluation of abundance by grouping into three groups (−, +, ++). If it is possible to evaluate the abundance of each species precisely with real numerical data, then it will be possible to perform statistical analyses to test if the correlation fits in with the Eq. (1) and to test the validity of Konno’s model. At present, however, it is very difficult to evaluate the abundance of each species in the field precisely and fairly with real numerical data. Measuring the frequency of egg finding may be an easy and efficiently way to estimate the abundance of each species qualitatively (e. g. grouping into ++, + , −) as shown in the present study. Even with this method, however, the frequency of egg finding may be affected by the structures of plant surfaces (simple leaf surface, complicate flower surface, colours of plant surface), environments (darkness, weather), searchers’ skills, etc., and therefore, fair and quantitative evaluation of abundance with precise numerical data is still very difficult. The qualitative categorization into three classes, very abundant ++, abundant + , and non-abundant −, based on egg-finding frequency (and not more than three classes) is what I can be confident of at present. Similar thing can be said to the quantitative evaluation or measurement of migratoriness. Future studies with fair and accurate qualitative numerical evaluation of abundance (and also migratoriness) and sophisticated statistical analyses will clarify with more certainty whether the correlation between G_h_ and abundance (or migratoriness) is valid, whether the correlation fits in with the Eq. (1), and whether Konno’s food web model^7^ is valid.

A further observation that supports the supposition that G_h_ correlates with abundance comes from a comparison between *P. rapae* and its sibling species, *Pieris melete* (Fig. 1). Unlike the worldwide pest *P. rapae*, *P. melete* is confined to East Asia, including Japan, Korea, and China, is less migrant, and is not regarded as a pest of cabbage, although *P. melete* lays eggs, feeds and grows normally on a variety of plants belonging to Brassicae, including cabbage^36–38^. The larval G_h_ of *P. melete* feeding on cabbage is moderate (0.728) and is significantly smaller (t-test, *p* < 0.0001) than the larval G_h_ of *P. rapae* feeding on cabbage (1.161). Under an equilibrium condition between herbivores (e.g., caterpillars) and carnivores (the natural enemies of caterpillars), the Konno’s food web model^7^ predicts that the biomass of carnivores, **c**, will be Eq. (2).

Then the top-down effect on herbivores by carnivores, **T**_**h**_ (/day), which indicates the probability that the herbivore biomass will be consumed by carnivores each day under equilibrium, could be expressed as

5 $${\mathbf{T}}_{{\mathbf{h}}} = { }{\mathbf{V}}_{{\mathbf{c}}} {\text{SP}}_{{{\text{hc}}}} = \frac{{\text{c}}}{{{\text{n}}_{{\text{c}}} }}{\text{ SP}}_{{{\text{hc}}}} = {\text{ G}}_{{\text{h}}} \;\; \left( {/{\text{day}}} \right),$$

where **V**_**c**_ (a ratio without a unit) stands for the volume of carnivore in a unit volume ecosystem in equilibrium.

**Figure 3:**
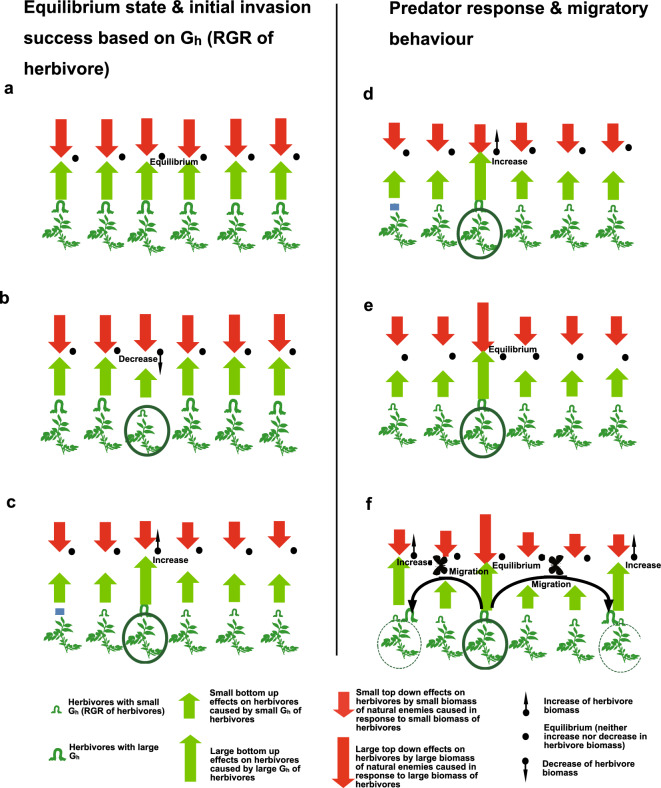
Diagram showing the ecological consequences of large G_h_ (RGR of herbivore). (**a**) When herbivores with a certain G_h_ start to feed on plants, the biomass of the herbivores will increase, but so will the biomass of their natural enemies (carnivores), causing the increase in herbivore biomass to be stopped at equilibrium. This means that the bottom-up effect from the G_h_ and the top-down effect from the presence of carnivores will equilibrate. The larger the G_h_ is, the larger the biomass of herbivores and the top-down effect at equilibrium will be. (**b**, **c**) Competitiveness of herbivores determined by their G_h_s. (**b**) The case of an herbivore with small G_h_ locally invading the area (solid circle) where an herbivore with large G_h_ already exists globally with carnivores in equilibrium. The top-down effect of carnivore, which is in equilibrium with the large G_h_, is expected to be larger than the bottom-up effect of the small G_h_ of an invader, and therefore, the invader with small G_h_ will decrease in biomass, fail to invade, and become competitively excluded by the herbivore with large G_h_. (**c**) The case of an herbivore with large G_h_ locally invading the area (solid circle) where an herbivore with small G_h_ already exists globally with a carnivore in equilibrium. The top-down effect of a carnivore that is in equilibrium with the small G_h_, is expected to be smaller than the bottom-up effect of large RGR of invader, and therefore, the invader with large G_h_ can increase in biomass and succeed in invasion. (**d–f**) Development of a migratory nature in an herbivore with large G_h_. (**d**) When an herbivore with a large G_h_ locally invades an area (solid circle) with an existing herbivore for whom the G_h_ is not high globally, the herbivore with large G_h_ will be able to increase its biomass at first, because the top-down effect will be globally small. (**e**) Soon, the biomass of the carnivore will increase and the top-down effect will also increase to equilibrate with the large G_h_ locally in the region where the herbivore with high G_h_ invaded (solid circle), with the result that the biomass of the herbivore can increase no further. (**f**) A herbivore with large G_h_ can still increase its biomass if it migrates from originally-invaded area (solid circle) to the surrounding areas (dotted circles) where the top-down effect is still small. Therefore, fitness increases when an herbivore with large G_h_ develops migratoriness.

Equations (2) and (5) mean that where an herbivore species with large G_h_ (G_h(large)_) comes into an ecosystem and lives for certain period of time, the amount of natural enemies will become proportionally large, and the top-down effect will increase until it finally reaches **T**_**c(large)**_, which equals G_h(large)_ in equilibrium (Fig. 3a). It can be said that the G_h_ of the ambient herbivores determines the strength of the top-down effect at a locality, which can be called the top-down effect field. Then, it is hard for an herbivore species with small G_h(small)_ to come in and become established in an area that is already inhabited by an herbivore species with large G_h(large)_, provided both herbivore species share natural enemies in common. This is because that the herbivore species with small G_h_ (G_h(small)_) can only tolerate a small amount of natural enemy or small top-down effect** T**_**h(small)**_ equal to G_h(small)_ to maintain equilibrium, and when the herbivore species with a large G_h(large)_ preexists, the large top-down effect **T**_**h(large)**_ = G_h(large)_ by natural enemies will be formed in equilibrium with the herbivore species with a large G_h(large)_, and this large top-down effect **T**_**h(large)**_ = G_h(large)_ will force the herbivore species with smaller G_h(small)_ into extinction, even when plant resources are abundant for both species (Fig. 3b). In opposite, it should be easy for an herbivore species with large G_h(large)_ to come in, increase in number, and become established in an area that is already inhabited by an herbivore species with small G_h(small)_, because the top-down effect of natural enemy in an area that is already inhabited by an herbivore species with small G_h(small)_ is expected to be low (**T**_**h**_ = G_h(small)_) there, and the herbivore with large G_h(large)_ should be able to increase in biomass (Fig. 3c). This scheme shows that it is likely that the herbivore species with large G_h_ can outcompete the herbivore species with small G_h_, and competitive exclusion takes place if they share a similar or identical set of natural enemies in common, even when plant resources are abundant and enough for both species. If two herbivore species are phylogenetically close to each other, it is likely that both species will share both specialist and generalist natural enemies in common. However, even when two of multiple herbivore species existing at the same locality are not necessarily close to each other phylogenetically, it is expected that those herbivore species will still share generalist natural enemies such as predators in common, and it is expected that those herbivore species will still tend to show some degree of competitive exclusion, even if the food resources are abundant. In regard to *P. rapae* and *P. melete*, they are sibling species very similar to each other physiologically and in appearance, and both feed on *Brassicae* plants (Fig. 1a–d). They share most natural enemies in common, including generalist natural pests such as birds, frogs, wasps, spiders, ants, and most specialist enemies such as parasitoid flies, with the exception of a parasitoid wasp, *Cotesia gromerata*, which is a major parasitoid of *P. rapae* but generally cannot parasitize *P. melete*^39^. However, with the great difference of larval G_h_ (and G_h_ throuout whole life stages), *P. rapae* can certainly outcompete the *P. melete* from cabbage field, and the model calculation predicts that *P. melete* will be able to compete with *P. rapae* only when *C. glomeriata* parasitize more than 90% of *P. rapae* larvae, and the actual G_h_ values of *P. repae* and *P. melete* are equivalent at the population level when considering the 90% biomass loss through mortality caused by *C. gloreriata* specifically on *P. rapae* and 10% surviving (i. e. if (0.1 × 208.5 = 20.85 is substituted into the Eq. (4) as pupal mass in place of original 208.5 in calculating larval G_h_ of *P. rapae*, the calculated larval G_h_ nearly equals that of *P. melete* (= 0.728)). However, previous studies at various sites, locations, and conditions show that the annual average of parasitism is around 81% under conditions that allow *P. rapae* to survive year round^40^. The parasitism rate exceeds 90% only in summer in Japan, and it is much lower (20–40%) where *P. rapae* is newly established^40^. This indicates that *P. rapae* outcompetes *P. melete* and exclusively serves as a pest on cabbage under most conditions, and competitive exclusion takes place even if food plants are abundant and is not a limiting factor for those herbivore species. Interestingly, on some other Brassicae plants, such as *Orychophragmus violaceus*, *Cardamine occulta*, and *Rorippa indica*, on which *P. melete* feed and which are found in natural habitats alone or together with *P. rapae*, the larval G_h_ of both *Pieris* species are much closer or almost the same, although that of *P. rapae* is still slightly larger than that of *P. melete*, and the model calculation indicates that under the moderate to high rates of parasitism by *C. glomeriata* that are often observed under field conditions, *P. melete* can even outcompete *P. rapae* (unpublished data). Thus, the scheme based of Konno’s model predicts that the herbivore species with larger G_h(large)_ will not only become more abundant but will also become more competitive than the herbivore species with small G_h(small)_ and will finally compete out the species with small G_h(small)_ from the habitat (Fig. 3b, c). Above view tells that it is advantageous for herbivorous insects to realize large G_h_, or grow fast, on a certain hostplant, and that there will be a strong selective pressure on herbivorous insects to strive for large G_h_, or more favorably, the maximum or the largest G_h_ among the insect herbivores feeding on a particular hostplant species in the same habitat; if not, the insect herbivore species will be competed out and will go to extinction. This means that to be a successful herbivore, the herbivorous insect species should not only develop the ability to grow on a certain hostplant, but also should develop ability to grow fast (large G_h_) or even should be one of the fastest growers with largest G_h_ or one of the best performers on the hostplant in the same habitat. Therefore, it is suggested that G_h_ is an important determinant factor of host plants of herbivores out in the field ecosystem. It is also suggested that the fact that a certain herbivore can grow and survive feeding on a certain plant species in laboratory conditions does not necessarily mean that the herbivorous species can survive in the wild on that particular plant species.

The statistical analyses of the data shown in Table 1 further revealed the general correlation between large larval G_h_ (RGR in larval stage) and migratoriness of herbivorous insect species (Fig. 2b). The scheme based on Konno’s food web model can explain why herbivores with large G_h(large)_ tend to be migratory (Fig. 3d–f). If herbivores with large G_h(large)_ stay in one place for a long time, then the biomass of carnivores and the top-down effect **T**_**h**_ of their natural enemies, including both specialist natural enemies such as parasitoids and generalist natural enemies such as predatory birds, insects and reptiles will increase locally up to G_h(large)_ (Fig. 3d, e). Such local increase in the top-down effect will, then, make it advantageous for herbivores with large G_h(large)_ to leave such regions and migrate to others that have a low top-down effect (**T**_**h**_ = G_h(small)_) from natural enemies (Fig. 3f), thus predicting that herbivores with large G_h_ tend to be migratory. The advantage of migration does not apply to herbivore with small G_h_. This is because herbivores with small G_h_ cannot compete with herbivores with large G_h_ due to the top-down effects caused by ubiquitous generalist natural enemies such as predatory birds, spiders, insets (ants, etc.), reptiles and amphibians common to both herbivores with small and large G_h_, it is suggested that the herbivores with small G_h_ tends to live in habitats free from herbivores with large G_h_. Typically, the habitats where only hostplants for the herbivore with small G_h_ is present and the hostplants for herbivores with large G_h_ is absent fulfil this condition. Then, for herbivore with small G_h_ to leave and migrate out of such favourable habitats without herbivores with large G_h_ means to compete with other herbivores with large G_h_, and is not adaptive, and therefore, it is not likely that migratoriness develops in herbivores with small G_h_. Above discussion tells why herbivore with larger G_h_ tends to be more migratory than those with smaller G_h_. The results in Fig. 2b clearly support this tendency.

Moreover, the above discussion about RGR of herbivores (G_h_) could help to clarify why groups of traits such as high growing speed and migratoriness tend to coexist, which has sometimes been discussed in the context of r/K strategies^41,42^. Both the coincidence of larval large G_h_ and migratoriness (Fig. 2b) and the scheme based on the Konno model (Fig. 3d–f) indicate that a high RGR in an herbivore (G_h_) is the primary cause of a migratory nature, and these frequently coinciding traits are not something like strategies, but rather a simple cause and effect relationship.

**Figure 4:**
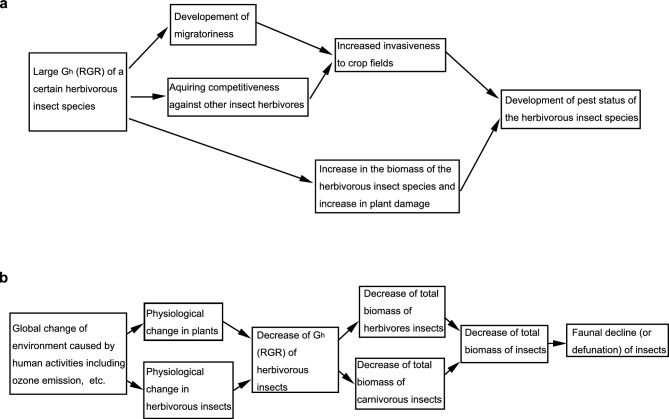
Possible roles of G_h_ (RGR of herbivorous insects) in the development of pest status of herbivorous insects and in the faunal decline of insects. (**a**) Mechanism of development of pest status in herbivorous insects caused by large G_h_. (**b**) Mechanism of faunal decline (or defaunation) of insects, a global change in ecosystem, caused by human activities including ozone emission etc., through the decrease of G_h_, a modification of plant–herbivore interface in the bottom of the food web.

The present results show that large G_h_ is critically important for an insect herbivore species (e.g. *P. rapae*) to obtain a pest status (Fig. 4a). As for *P. rapae*, first, large G_h_ (1.161) made *P. rapae* migratory and facilitate *P. rapae* to fly into a newly established cabbage field. Second, the large G_h_ makes *P. rapae* competitive and enables *P. rapae* to outcompete its competitors and occupies cabbage field exclusively; the RGR of an herbivore (G_h_) is an important factor that determines the competitiveness of species. For example, the larval G_h_ of *P. melete* feeding on cabbage is 0.728, rather large for an herbivorous species (Table 1), but in the cabbage field, *P. melete* still cannot compete with *P. rapae*, which has a much higher larval G_h_ (1.161) when feeding on cabbage. In this case, as both species share the same (or similar) habitats, host plants, or natural enemies, and *P. melete* is excluded from the cabbage field. Thus, Large G_h_ makes herbivorous insects invasive through development of migratoriness and competitiveness. Third, once *P. rapae* succeeded in invasion to the cabbage field, large larval G_h_ further allows *P. rapae* to become abundant even under the existence of efficient natural enemy. Therefore, the large G_h_ is critically important for an insect herbivore species to become a serious pest, through the development of migratoriness and competitiveness, which together make the species invasive, and through abundance (Fig. 4a). For a herbivore to be regarded as a pest, it should not only become abundant and inflict high-level damage to crops, but it should also migrate to newly established crop fields. A high RGR in a herbivore (G_h_) is a critically important factor in terms of allowing a herbivore to fulfil all the three of these conditions. The present study showed the facts that *P. rapae*, which has an extremely large larval G_h_, has become one of the world’s most serious pests, with a capacity for causing unusually severe damage to plants, and that *Lampides boeticus*, which has the second largest larval G_h_ in Table 1 following that of *P. rapae* is a serious and very abundant migratory pest of legume worldwide including Japan and India^27^. These facts suggested that a high RGR of herbivorous insect on a particular plant species is an important factor determining whether a herbivore will become a pest. Indeed, large (larval) G_h_ might be suitable for use as an index to predict the likeliness of a certain insect herbivore to become a pest of a certain plant species. On the contrary, decreasing the (larval) G_h_ of the pest herbivore by certain methodology (use of anti-nutritive factors, antifeedants, decreasing nutritive value of plant by fertilizing methods or by selection of cultivars, etc.) is suggested to be very effective to relieve the pest-status of insect herbivores and plant damage.

The present study revealed the importance of high RGR of insect herbivores, G_h_, as very important ecological factor that determines ecological traits of insect herbivores including abundance, migratoriness, competitiveness, and pest status of insect herbivores. However, G_h_ is not the only factor that affects the development of these traits. The Eq. (1) of Konno’s food web model^7^ shows biomass of herbivore **h** is not only a function of G_h_, but also a function of S (searching abitity of carnivore/predator) and P_hc_ (probably of carnivore /predator to capture herbivore), both of which are indicators of efficiency of predators and/or evasiveness of herbivores from predators. This means that the efficiency of predators and/or predator evasiveness of herbivores are also important determinant factors of herbivore abundance. My result showed that, although it is a general rule that insect herbivores with large G_h_ tend to be abundant and /or migratory, there are exceptions. For example, although both *Byasa alcinous* (Papilionidae) (larval G_h_ = 0.392) and *Mamestra brassicae* (Noctuidae) (larval G_h_ = 0.380) show relatively small larval G_h_, they are abundant. *Byasa alcinous* (Papilionidae) (larval G_h_ = 0.392) feeds on toxic *Aristolochia debilis*, and sequesters the toxic component that is unpalatable to natural enemies such as birds^33^, while *Mamestra brassicae*, a major pest of cabbage and other crops^34^, stays underground in the daytime^35^, presumably to escape from predation by natural enemies, and both herbivore species developed predator evasiveness. Another exceptional species is *Anthochalis scolymus*, which has a very large larval G_h_ (G_h_ = 0.955) that is close to larval G_h_ of highly abundant *P. rapae*, (larval G_h_ = 1.161), but is not very abundant. Different from multivoltine *Pieris rapae*, this species, together with other *Anthochalis* species, are strictly monovoltine and have extremely long diapause, one year or more as pupae, and the population and biomass loss during the long diapause may possibly explain why *Anthochalis scolymus* is not very abundant. The discrepancy of the prediction by Konno’s model and the result in *Anthochalis scolymus* may possibly arise from the structure or assumption of the Konno’s model, i.e., Konno’s model is equilibrium-based model, which assume ecological conditions to be constant through time course, but in the case of *A. scolymus*, a monovoltine species, ecological conditions are not constant. Multivoltine insects in tropical ecosystems meet such conditions (constant conditions) well, but temperate monovoltine ecosystems do not meet such conditions well. Therefore, it is suggested that Eq. (1) predicts the abundance of herbivore more precisely in tropical multivoltine ecosystems rather than temperate monovoltine ecosystems. Further, if we want to be strict to the original Konno’s model, average RGR of herbivore, G_h_ through whole generation stages (egg, larva, pupa, adult) or RGR of population through whole stages should be used. But, the methodology used in the present study used larval G_h_ (RGR in larval stage) as a substitute (or approximation) of RGR of population. This is because in herbivorous insects such as lepidopteran insects, larval stage is growing stage under severest top-down pressure compared to other stage and most of bottom-up and top-down processes important in determining biomass of herbivore are supposed to be observed in the larval stage; eggs and pupae are non-growing stages and not easily found by predators because of their immobile and cryptic nature and adults are non-growing stage and not under high top-down pressure compared to larval stage because the high mobilities of adults enable adults to escape from predation. Therefore, estimating and calculating G_h_ using data only from larval stage seems not only to be a simple and convenient way to determine G_h_, but also seems to be a good approximation of G_h_ through whole stages; the egg and the larval stages can be regarded as stages that the bottom-up and top-down processes are minimal and these stages can be neglected. However, as in the case of *Anthochalis scolymus* with a prolonged pupal stage (diapause stage) that last for nearly a year, the accumulated top-down effect or mortality during very long non-larval stage cannot be neglected. Therefore, the methodology presented in this study to predict the abundance of insect herbivores using the data from the larval stage or larval G_h_ is very valid against multivoltine insects in stable ecosystem such as tropical rainforests, but the methodology has potential limitation in highly unstable and/or seasonal ecosystem with prolonged diapause or non-growing periods. It also means seasonality and voltinism have effects on determining the abundance of herbivorous insects apart from the efficiency of predators and / or predator evasiveness of herbivorous insects and G_h_ of herbivores insects.

Further, although G_h_ (RGR of herbivore) is a factor that characterizes the plant–herbivore interface at the bottom of the food web, both the example with *P. rapae* and Konno’s food web model suggest that G_h_ is an important basic determinant factor affecting the whole ecosystem, including animal abundance (both herbivores and carnivores), fauna size, and the levels of plant damage (Fig. 4b). In recent years, a significant decline of insect fauna (i.e., a significant decrease in the total combined biomass of herbivorous and carnivorous insects; defaunation) has been reported and has attracted a great deal of public attention^43,44^. The present study suggests that changes to the plant–herbivore interface as a result of human activities and the subsequent decrease in the G_h_ may be at least partly responsible for this faunal decline via reductions in the biomass of both herbivores and carnivores. For example, a recent report showed that herbivorous insects grow more slowly on plants grown under high ambient concentrations of ozone^45–47^. Since ozone and related NOx are produced by human activities and their concentrations are increasing ubiquitously around the world, and at present, 30–70% increase in ozone concentration compared to 1896–1975 are observed even in rural and polar areas in northern hemisphere^47^, it is possible that the recent faunal decline (or defaunation) of insects^43,44^ is partly attributable to ozone emission or other human activities through a decrease in G_h_ (Fig. 4b). In opposite, it is also possible that consequences of human activities such as increased nitrogen in environment may improve the nutritive quality of plant to herbivorous insect, increase G_h_ of herbivores (or pest insects), and then increase the biomass of herbivores (or outbreak of pests), and finally increase the damage of plants by herbivorous (or pest) insects. In either case, the change in G_h_ (RGR of herbivorous insect) is a good indicator to predict the change in ecosystems caused by human activities.

## Conclusion

The contributions of the present study can be summarized as follows. First, an Eq. (4) was proposed for the standardized measurement of larval G_h_ (RGR of larvae of herbivorous insects), which is shown to be a distinct and diverse measure among herbivore–plant pairs (Table 1). Second, the extraordinarily high larval G_h_ of *P. rapae* was shown to account for the global pest status of *P. rapae* (Table 1), and larval G_h_ was shown to correlate with abundance (Fig. 2a) and migratoriness (Fig. 2b) of herbivores and to be an important ecological factor that determines and/or predicts the abundance of herbivores, the degree of herbivory and plant damage, the migratory nature of herbivores, and the competitiveness (or invasiveness) of herbivore species against other herbivore species, all of which are important for an insect species to become a pest. Reasonable explanations of the underlying mechanisms were also provided based on a mathematical model. Finally, the present results suggest that human activities, whether intentionally or not, may have changed the G_h_ (RGR of herbivores) at the bottom of the food web, and that this could have a major impact on the whole ecosystem, including through a decline of fauna (defaunation), extinction or outbreak of animal species, and changes in the populations of various pests.

## Methods

### Insects

Eggs of the following species were collected from host plants in the wild in Tsukuba, Ibaraki, Japan (36° N, 140° E): *Pieris rapae* (Pieridae), *Eurema mandarina* (Pieridae), *Colias erate* (Pieridae), *Anthochalis scolymus* (Pieridae), *Papilio xuthus* (Papilionidae), *Papilio Memnon* (Papilionidae), *Papilio machaon* (Papilionidae), *Byasa alcinous* (Papilionidae), *Graphium sarpedon* (Papilionidae), *Lethe sicelis* (Nymphalidae, by following adults laying eggs), *Lampides boeticus* (Lycaenidae), *Curetis acuta* (Lycaenidae), *Spodoptera litura* (Noctuidae), *Theretra oldenlandiae* (Sphingidae), *Theretra japonica* (Sphingidae), *Acosmeryx castanea* (Sphingidae), *Macroglossum pyrrhostica* (Sphingidae), *Neogurelca himachala* (Sphingidae), and *Agrius convolvuli* (Sphingidae). Eggs of *Mamestra brassicae* (Noctuidae) were collected from the leaves of cabbage cultivated in the field in Sapporo, Hokkaido, Japan (43° N, 141° E) and kindly supplied by Dr. Kenji Takashino (NARO). Eggs of *Mycalesis gotama* (Nymphalidae) and *Papilio protenor* (Papilionidae) were collected from adults captured from the wild in Tsukuba, Ibaraki, Japan (36° N, 140° E) and those of *Pieris melete* (Pieridae) were collected from adults captured in Hokuto, Yamanashi, Japan (36° N, 138° E), by letting adults lay eggs on the host plants. Eggs of a line of the Eri silkmoth, *Samia ricini* (Saturnidae), were obtained from *S. ricini* (Saturniidae) adults maintained at our institute as experimental insects. Eggs of *Bombyx mandarina* were collected from a laboratory strain of *B. mandarina* originating from a female from Gunma, Japan and a male from Yamanashi, Japan, and were kindly supplied by Dr. Natuo Kômoto (NARO).

### Plants

As *Brassica oleracea*, a *Brassica oleracea var Capitata* (cabbage) cultivar Kinkei-201 (Sakata Seed Corporation, Yokohama, Japan) and a wild variety 0-176 that originated in Turkey and was kept and supplied by NARO Genebank, Japan were used in bioassays. As *Ipomoea batatas* (sweet potato), a cultivar Beniharuka that was developed by NARO, Japan was used. As *Daucus carota* (carrot), a cultivar Benikanade (Nanto Seed Corporation, Nara, Japan) was used. *Morus alba var Shinichinose* (mulberry tree), *Ricinus communis* (castor oil plant), and *Citrus sudachi* (sudachi orange) that were kept and grown in our institute (NARO, Tsukuba, Japan) were also used. Plant materials of all other plant species listed below were collected from the wild in Tsukuba, Ibaraki, Japan (36°N, 140°E) in areas owned by NARO, where there were no legal regulations limiting the collection. The plants were identified by Kotaro Konno and the voucher specimens with following ID numbers were deposited in the Institute of Agrobiological Sciences, National Institute of Agriculture and Food Research Organization (NARO); *Lespedeza cuneata var. cuneata* (SH-22-KK-01), *Trifolium repens* (white clover) (SH-22-KK-02), *Trifolium pratense* (red clover) (SH-22-KK-03), *Cardamine occulta* (SH-22-KK-04), *Aristolochia debilis* (pipevine) (SH-22-KK-05), *Cinnamomum camphora* (camphor tree) (SH-22-KK-06), *Causonis japonica* (bushkiller) (SH-22-KK-07), *Paederia foetida* (skunkvine) (SH-22-KK-08), *Solanum carolinense* (Carolina horse nettle) (SH-22-KK-09), *Pleioblastus chino var. chino* (small monopodial bamboo) (SH-22-KK-10), *Miscanthus sinensis* (Chinese silvergrass) (SH-22-KK-11), and Pueralia montana var. lobata (kudsu vine) (SH-22-KK-12).

### Measurement of herbivory on cabbage by the small white butterfly larvae

The seeds of cabbage (*Brassica oleracea*, Kinkei-201 and 0-176) were sown in pots and kept in a sunroom under 25 °C. 25 days later, the cabbage seedlings were planted out in a fertilized field or in a forest in Tsukuba, Ibaraki, Japan (36° N, 140° E) in late May–early June period or in late September. At 20–49 days after planting, leaf damages (% leaf area) were measured. Experimental research and field studies on plants were performed under the guidelines of the National Agriculture and Food Research Institute (NARO), Japan, to which the author belongs.

### Measurement of larval G_h_ (average RGR of herbivores during the whole larval stage) and analyses of relationship between larval G_h_ and ecological traits of herbivorous insects such as abundance and migratoriness

The egg masses of the following insect species, whose eggs have relatively large mass (ca. 0.3 mg or larger) and are easy to detach from leaves without breaking, were measured using an analytical balance (Mettler Toledo, AB204-S; readability and repeatability: 0.1 mg): *Papilio xuthus*, *Papilio protenor*, *Papilio Memnon*, *Papilio machaon*, *Byasa alcinous*, *Graphium* *sarpedon*, *Theretra oldenlandiae*, *Theretra japonica*, *Acosmeryx castanea*, *Macroglossum pyrrhosticta*, *Neogurelca himachala*, *Agrius convolvuli*, *Bombyx mandarina*, *Samia ricini*, *Lethe sicelis*, and *Mycalesis gotama*. The egg masses of the following insect species, whose eggs have relatively small mass (less than 0.3 mg) and are difficult to detach from leaves without breaking, were estimated by measuring the size of the eggs: *Pieris rapae*, *Pieris melete*, *Spodoptera litura*, *Mamestra brassicae*, *Eurema mandarina*, *Colias erate*, *Anthochalis scolymus*, *Lampides boeticus*, *Curelis acuta*, and *Henosepilachna vigintioctopunctata*. The hatched larvae were fed excised young but mature leaves—i.e., leaves that had just reached their mature size but were still young and soft—at 25 °C with a 16-h-light/8-h-dark photoperiod. Leaves were replaced with fresh ones every other day until pupation. The mass of pupae was measured one day after the pupation using an analytical balance (Mettler Toledo, AB204-S). Larval G_h_ was calculated as follows using the pupal mass, egg mass, and larval-stage duration (days) in Eq. (4).

Larval G_h_ = exp_10_ [ log_10_ {pupal mass / egg mass} / {duration (days) of larval stage}] -1    (/day)   (4)                                                                                                                                                                                                                                                                                                                                                                                                                                                                                     This method for measuring and calculating larval G_h_ was developed herein and succeeded in yielding consistent, reliable, and comparable larval G_h_ values from the mass of eggs and pupae. Unlike in previous studies, the mass of larvae was not employed, since larvae can contain a large (up to 80%) and variable amount of food materials and digestive fluid in their digestive tracts, which can be a significant source of fluctuation.

Herbivorous insect species on hostplants are categorized based on abundance and migratoriness. The abundance of herbivorous insect of hostplants is judged by the abundance (density) of eggs on hostplants and/or easiness to find eggs on the hostplant; Typically, if more than 10 eggs of an herbivorous insect species are found in 10 min on the surface of the hostplant by a person, then the herbivorous insect species is categorised as very abundant (abundance ++), if less than 10 eggs in 10 min but more than 10 eggs per hour are found, then the herbivorous insect species are categorized as abundant (abundance +), and if less than 10 eggs per hour are found, the herbivorous insect species are categorized as non-abundant (abundance −). Herbivorous insect species that have reported to be migratory, that invaded, that are found ubiquitously in remote islands, and/or that are frequently reported from areas out of its original rages are categorized as migratory (migratoriness +)^13,14,25–31^. For the detail results of categorization, see Table 1.

Relationship between larval G_h_ and ecological traits such as abundance and migratoriness was analyzed by calculating and comparing the average and SD of larval G_h_ for insect herbivore species belonging to each category, abundance (++, +, −) and migratoriness (+, −), and then performing both non-parametric (Mann-Whitney *U*-test, two-sided) and parametric (Student’s *t*-test, Tukey’s test for multiple comparisons, two-sided) statistical analyses.

## Data Availability

The datasets generated during and/or analysed during the current study are available from the corresponding author on reasonable request.
